# Conjugating a groove-binding motif to an Ir(iii) complex for the enhancement of G-quadruplex probe behavior†
†Electronic supplementary information (ESI) available: Compound characterisation and supplementary data. See DOI: 10.1039/c6sc00001k


**DOI:** 10.1039/c6sc00001k

**Published:** 2016-01-28

**Authors:** Modi Wang, Zhifeng Mao, Tian-Shu Kang, Chun-Yuen Wong, Jean-Louis Mergny, Chung-Hang Leung, Dik-Lung Ma

**Affiliations:** a Department of Chemistry , Hong Kong Baptist University , Kowloon Tong , Hong Kong , China . Email: edmondma@hkbu.edu.hk; b State Key Laboratory of Quality Research in Chinese Medicine , Institute of Chinese Medical Sciences , University of Macau , Macao , China . Email: duncanleung@umac.mo; c Department of Biology and Chemistry , City University of Hong Kong , Kowloon Tong , Hong Kong , China; d University of Bordeaux , ARNA Laboratory , Bordeaux , France . Email: jean-louis.mergny@inserm.fr; e INSERM , U869 , IECB , Pessac , France

## Abstract

In this study, the reported G-quadruplex groove binder benzo[*d*,*e*]isoquinoline was linked to a cyclometallated Ir(iii) complex to generate a highly selective DNA probe **1** that retains the favorable photophysical properties of the parent complex. The linked complex **1** showed advantages of both parent complex **2** and groove binder **3**. Similar to **3**, the conjugated complex **1** exhibits a superior affinity and selectivity for G-quadruplex DNA over other conformations of DNA or proteins, with the fold enhancement ratio obviously improved compared with parent complex **2**. The molecular modelling revealed a groove-binding mode between complex **1** and G-quadruplex. Meanwhile **1** also possesses the prominent advantages of transition metal complex probes such as a large Stokes shift and long lifetime phosphorescence, which could be recognized in strong fluorescence media through time-resolved emission spectroscopy (TRES). We then employed **1** to develop a detection assay for AGR2, a potential cancer biomarker, as a “proof-of-principle” demonstration of the application of a linked complex for DNA-based detection in diluted fetal bovine serum. We anticipate that this conjugation method may be further employed in the development of DNA probes and have applications in label-free DNA-based diagnostic platforms.

## Abstract 2

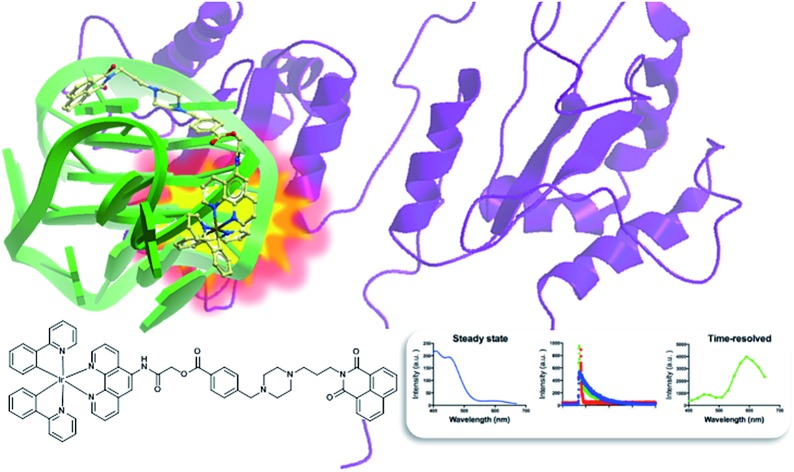
G-quadruplex groove binder benzo[*d*,*e*]isoquinoline was linked to a Ir(iii) complex to generate a highly selective DNA probe.

## Introduction

The anterior gradient homolog 2 (AGR2) protein plays an important role in the proliferation and migration of tumor cells,^1^ and the detection of AGR2 is very useful in cancer diagnosis. The common lengthy duration of measurement, which requires several days for sample translation and analysis, can increase the mental burden on patients during testing,^2^ prompting a need for alternative detection methods. Oligonucleotide-based detection has received wide attention in the recent literature.^3^–^21^ It depends on the ability of an oligonucleotide to change conformation upon exposure to the target. The new conformation can be converted into a measurable signal by appropriate signal transducers. One recent example is an oligonucleotide-based detection assay for AGR2 using fluorescently labeled DNA.^2^,^22^ In this assay, AGR2 induces a structural change of the DNA oligonucleotide, thus altering the distance between the fluorophore and quencher.^2^,^22^ This approach has the disadvantage that fluorescently labeled oligonucleotides are relatively expensive, thus a more economical alternative may possibly involve a label-free strategy that utilizes unmodified DNA and selective DNA probes. Several organic dyes have been reported as DNA probes, such as thioflavin T (ThT),^23^,^24^ crystal violet (CV)^25^ and thiazole orange (TO),^26^ some of which can recognize G-quadruplex (G4) DNA *via* the end stacking of a G-quartet.

More recently, transition metal complexes have been explored as selective probes for DNA, in particular the G-quadruplex DNA structure.^27^–^33^ Transition metal complexes have several useful qualities for this application. Their ligands can be varied in order to tune their photophysical properties and interactions with target biomolecules. Their phosphorescence generally has a long emission lifetime, which can be distinguished from highly fluorescent media through the use of time-resolved emission spectroscopy (TRES). Additionally, the large Stokes shifts of metal complexes can reduce self-quenching.^34^,^35^ Our group has previously reported a series of cyclometallated Ir(iii) complexes as selective G-quadruplex probes and demonstrated the optimization of their photophysical and G-quadruplex-binding properties *via* changing the auxiliary N^N or C^N ligands.^36^ However, as structural changes affect both the G-quadruplex-binding affinity and photophysical properties, it can sometimes be difficult to improve the G-quadruplex recognition abilities of a metal complex without adversely influencing its photophysical characteristics.

**Fig. 1:**
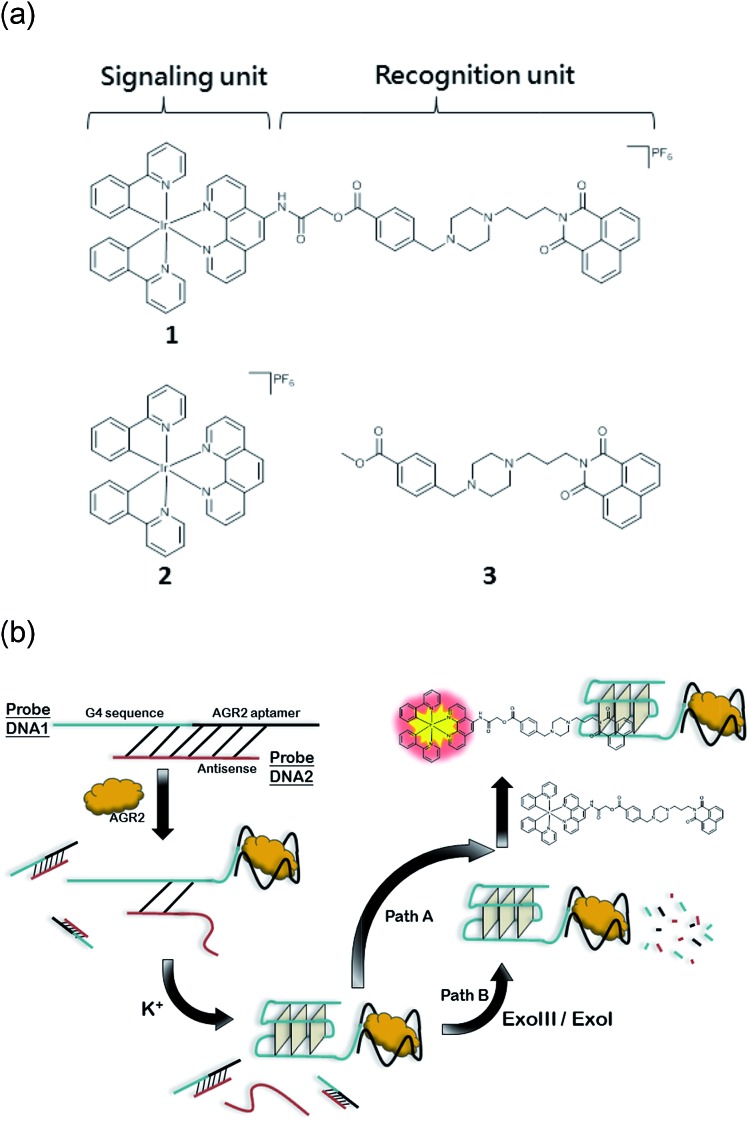
(a) Chemical structure of Ir(iii) complexes **1** and **2** and the G-quadruplex loop binder **3**. (b) Schematic diagram illustrating the AGR2 sensing platform utilizing the DNA binder linked Ir(iii) complex **1**.

In this study, we sought to design and synthesize a benzo[*d*,*e*]isoquinoline-linked Ir(iii) complex **1** by functionalizing a parent luminescent Ir(iii) complex **2** with a known G-quadruplex groove binder **3** (Fig. 1).^37^,^38^ The linked complex **1** can therefore be considered to be comprised of a signaling unit, the Ir(iii) complex **2**, linked to a recognition unit, the benzo[*d*,*e*]isoquinoline motif **3**. In our design, the recognition unit associates with G-quadruplex DNA, altering the environment of the probe. The signalling unit is sensitive to this change, and a corresponding change in the luminescence response can be measured. Thus, our desired outcome for complex **1** was to (1) localize the binding area of the metal complex to G-quadruplex DNA, thus increasing the selectivity, and (2) strengthen the binding affinity of the Ir(iii) complex for G-quadruplex DNA while maintaining its desirable photophysical properties. And finally, we applied Ir(iii) complex **1** to construct an AGR2 detection assay to demonstrate the proof-of-concept application of the Ir(iii) complex functionalized with a G-quadruplex binder.

## Results and discussion

To demonstrate the additional G-quadruplex binding affinity contributed by **3**, we chose the cyclometallated complex **2** as our parent complex, as **2** shows minimal selectivity for G-quadruplex DNA. As a G-quadruplex-based detection platform generally exploits the switching of DNA from ssDNA or dsDNA to G-quadruplex, the high G-quadruplex/ssDNA or G-quadruplex/dsDNA luminescence enhancement ratio of a G-quadruplex probe can increase the sensitivity of the detection platform. Also real biological samples usually contain matrix ssDNA or dsDNA as common interferences, thus a high enhancement ratio is crucial in real applications. Complex **2** displayed a *ca.* 1.3 to 1.5 luminescence enhancement ratio for ckit87up, ckit1 and Pu27 G-quadruplexes over double-stranded DNA, while a *ca.* 1.5 luminescence enhancement ratio over single-stranded DNA (ssDNA) was observed (Fig. S1a†). Moreover, a fluorescence resonance energy transfer (FRET) melting assay indicated that 5 μM of **2** did not stabilize F21T G-quadruplex DNA (Fig. S2†). This suggests that complex **2** binds weakly to the G-quadruplex motif, which could account for its low luminescence enhancement towards G-quadruplex DNA.

As all known G-quadruplex structures are characterized by grooves which are structurally and chemically very different from the minor groove of dsDNA, we decided to link a G-quadruplex groove-binding motif to parent complex **2**. In 2008, Ma and co-workers reported a drug-like G-quadruplex binding ligand found through high throughput virtual screening (VS).^39^ In the following year Randazzo and co-workers incorporated VS and NMR experiments to identify a G-quadruplex groove binder from 6000 compounds.^37^ Following this study, in 2012 our group identified the carbamide^40^ motif from a natural product and natural product-like compound database of over 20 000 compounds through VS. To our knowledge, there are few other examples of utilizing a structure-based virtual screening approach to screen a large number of compounds for G-quadruplex groove binders. As these scaffolds were screened from a large database, we decided to choose the recognition unit from these two studies. The benzo[*d*,*e*]isoquinoline scaffold **3** reported by Randazzo's group was chosen as the G-quadruplex recognition motif because it could be easily attached to the N^N donor ligand using a simple synthetic protocol. The selective G-quadruplex-binding of **3** was validated through FRET melting (Fig. S3†).

**Scheme 1:**
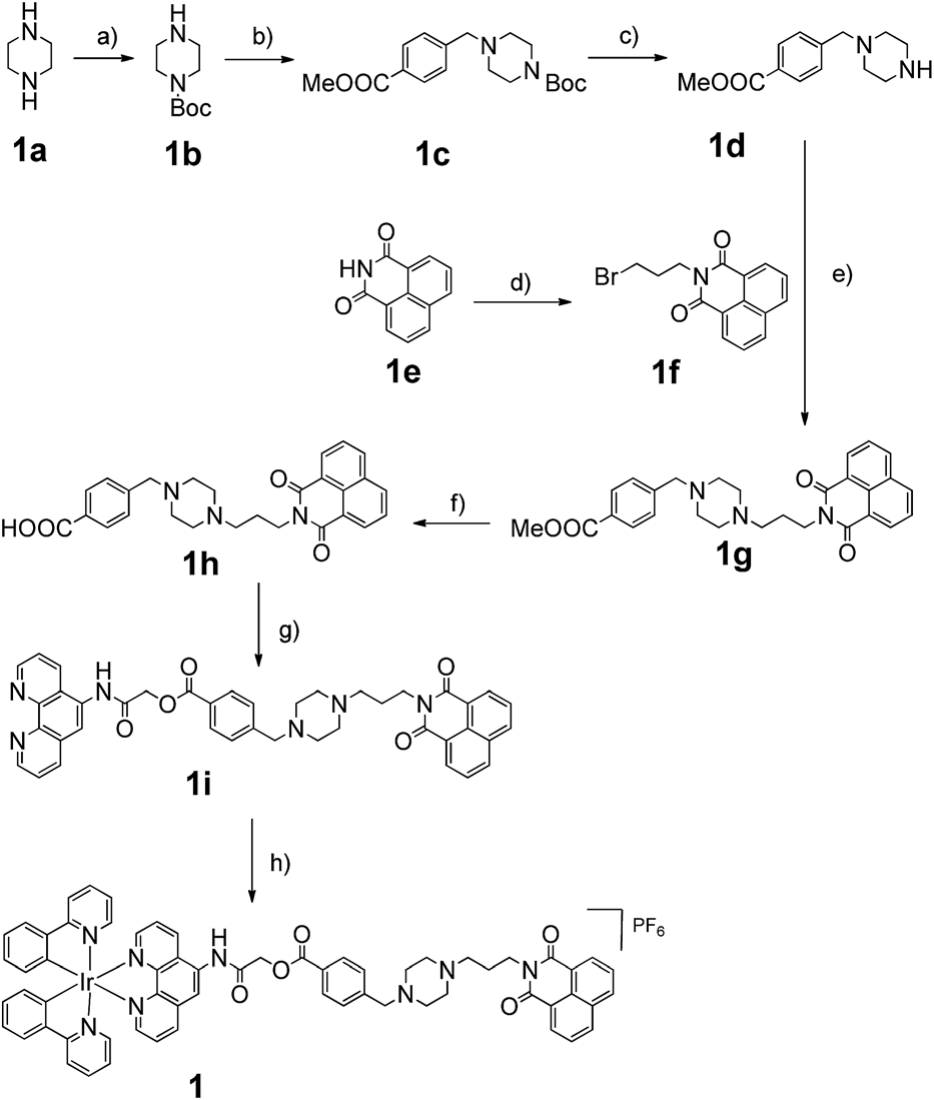
Synthesis of linked Ir(iii) complex **1**. Reagents and conditions: (a) Boc_2_O, CH_2_Cl_2_, 0 to 25 °C, 3 h, 76%; (b) methyl-4-(bromomethyl)benzoate, K_2_CO_3_, CH_3_CN, 0 to 25 °C, overnight, 91%; (c) TFA, CH_2_Cl_2_, 25 °C, overnight, 95%; (d) 1,3-dibromopropane, K_2_CO_3_, DMF, 40 to 50 °C, overnight, 76%; (e) K_2_CO_3_, DMF, 80 °C, 6 h, 72%; (f) LiOH, H_2_O, MeOH, reflux, 2 h, 99%; (g) 2-chloro-*N*-(1,10-phenanthrolin-5-yl)acetamide, K_2_CO_3_, DMF, 65 °C, overnight, 75%; (h) dichloro-bridged [Ir(ppy)_2_Cl]_2_, MeOH : CH_2_Cl_2_ = 1 : 1, 25 °C, overnight, 70%. DMF = *N*,*N*-dimethylformamide, Boc_2_O = di-*tert*-butyl dicarbonate and TFA = trifluoroacetic acid.

The Ir(iii) complex **1** was synthesized by the reaction of a precursor complex with a phenanthroline N^N ligand derivatized with **3**, as depicted in Scheme 1. The first step was the protection of piperazine **1a** with Boc_2_O to give **1b**, followed by treatment with methyl-4-(bromomethyl)benzoate to give intermediate **1c**. After deprotection of the Boc group with trifluoroacetic acid, the resulting compound **1d** was reacted with compound **1f**, which was obtained by the alkylation of naphthalimide **1e** with 1,3-dibromopropane, to afford derivative **1g** in 72% yield. After hydrolysis of the methyl ester with LiOH, the resulting acid **1h** was immediately reacted without further isolation with 2-chloro-*N*-(1,10-phenanthrolin-5-yl)acetamide to yield the 1,10-phenanthroline derivative **1i**. Finally **1i** was reacted with half an equivalent of the organometallated dimer [Ir(ppy)_2_Cl]_2_, followed by anion exchange with NH_4_PF_6_, giving the Ir(iii) complex **1** in 70% yield. The structures of the compounds were confirmed using NMR spectroscopy, elemental analysis and mass spectrometry (see the ESI†).

FRET-melting assays revealed that complex **1** selectively stabilizes G-quadruplex DNA. The melting temperature of F21T G-quadruplex DNA was increased by 13 °C upon the addition of 5 μM of **1** (Fig. S4a†), while no significant increase in the melting temperature was observed for dsDNA at the same concentration (Fig. S4b†). Furthermore, the presence of 10 μM of competitor DNA did not significantly affect the melting temperature enhancement of G-quadruplex DNA in the presence of **1** (Fig. S4c†). This result indicates that complex **1** exhibited a stronger affinity towards G-quadruplex than **2**, which is attributed to the attachment of the G-quadruplex-binding motif **3**.

In emission titration experiments, **1** displayed a *ca.* 3.5 luminescence enhancement ratio for ckit87up, ckit1 and Pu27 G-quadruplexes over ssDNA, while a *ca.* 3.2 luminescence enhancement ratio over dsDNA was observed (Fig. S1a†). This is comparable to organic G-quadruplex probes, which exhibited a high fold-enrichment for G-quadruplex DNA and moderate enhancement for ssDNA and dsDNA^23^,^41^ Meanwhile, modified ThT analogues have been reported by Kuwahara's group with high G-quadruplex/ssDNA or G-quadruplex/dsDNA enhancement ratios.^24^

**Fig. 2:**
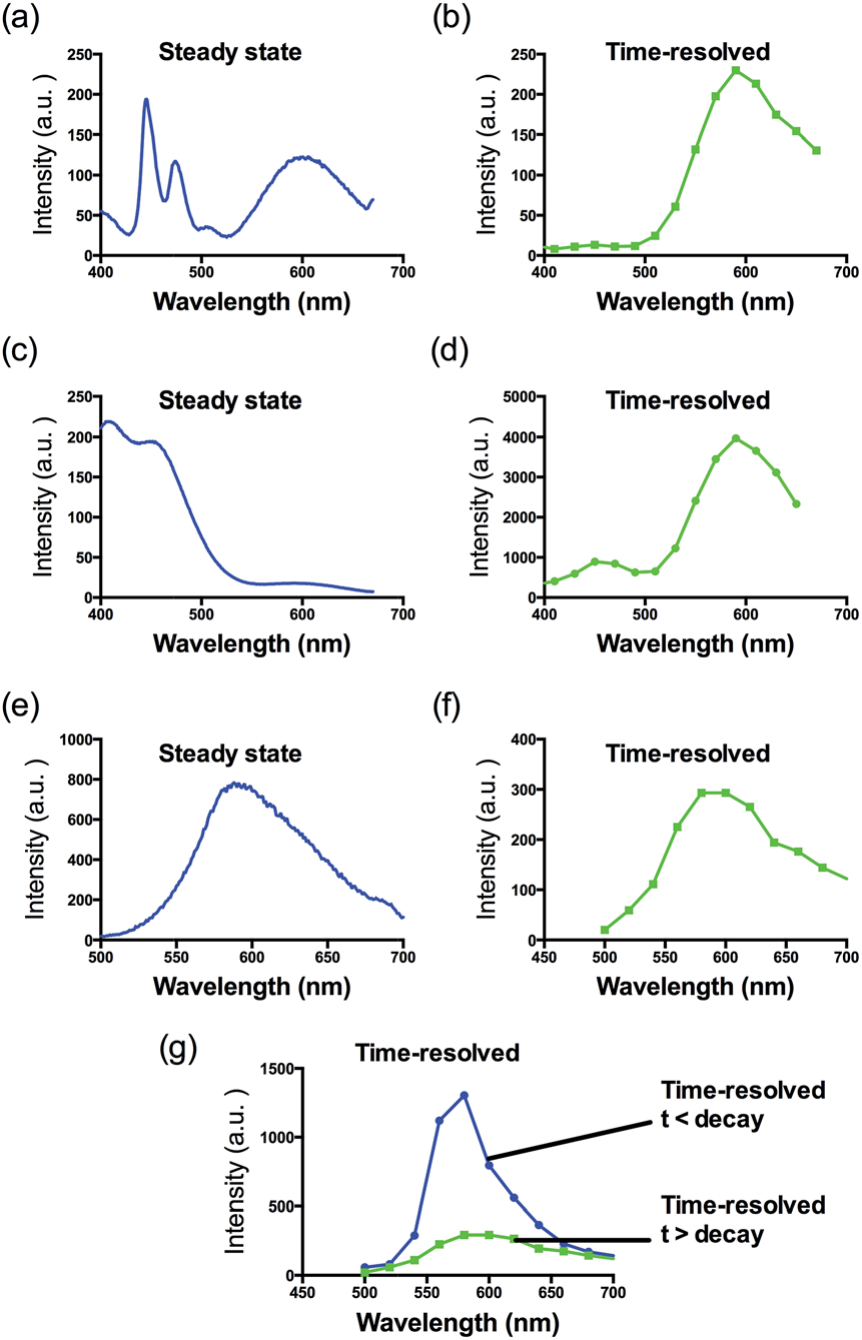
Steady-state photoluminescence and time-resolved emission spectra of **1** in the presence of (a and b) perylene, (c and d) coumarin and (e–g) rhodamine B fluorescent media.

Consistent with other Ir(iii) complexes, **1** displayed a *ca.* 240 nm Stokes shift which is 3.7-fold larger than that of ThT, and a microsecond lifetime (Table S2†) which in principle could distinguish the long lifetime luminescence of **1** from the high autofluorescence of the surrounding sample matrix environment using TRES. This is particularly important in the application of the sensing probe in real samples. In order to demonstrate that we could identify the luminescence of **1** in high autofluorescence samples, rhodamine B (RhB), coumarin (Cm) and perylene (PY) were introduced into **1** as sources of autofluorescence. In the steady-state emission spectra, PY displayed an emission peak located in the 450 nm region while the emission of **1** was observed clearly at 590 nm. Meanwhile, the strong peak of Cm is located at 420 nm with a tail that extends up to 650 nm. In this scenario, the peak of **1** is significantly influenced by the peak tail. Meanwhile, the RhB–**1** mixture displayed only one peak, presumably due to the similar peak maxima of RhB and **1**. For the TRES measurements, the time gate is defined as the time after the complete fluorescence decay of the three organic dyes. Upon TRES, no emission peak corresponding to Cm and PY was observed and the emission peak of **1** had become dominant. For the RhB–**1** mixture, the peak intensity was reduced upon TRES. We believe the reduced peak is attributed to **1** only, since the fluorescence of RhB was completely decayed before TRES (Fig. 2). The results indicate an advantage of our long lifetime luminescent complex **1**, namely that the luminescence of **1** could be identified using TRES measurements and potentially be applied in a strong autofluorescence sample matrix.

The superior luminescence and binding selectivity of **1** compared to the parent complex **2** demonstrates that the attachment of the G-quadruplex-binding benzo[*d*,*e*]isoquinoline motif successfully enhanced the interaction between the metal complex and G-quadruplex DNA. To examine the binding area of complex **1**, we investigated the luminescence response of complex **1** towards G-quadruplexes containing different size 3′-side loops (Fig. S5†). The G-quadruplex structure in this experiment has been investigated in the literature.^42^ The luminescence enhancement of complex **1** increased with loop size from 2 nucleotides (nt) and reached a maximum enhancement at 6 nt. This suggests that the G-quadruplex loop is involved in the binding interaction with **1**, which is consistent with the previous observation that motif **3** mainly recognizes the 3′-side of G-quadruplex grooves.^37^ Finally, **1** showed a weaker luminescence response towards an intermolecular G-quadruplex (5′-TG_4_T-3′) compared to an intramolecular ckit1 G-quadruplex, which we attributed to the lack of loops in an intermolecular G-quadruplex for recognition by **1**. Possibly however, all grooves in the tetramolecular complex are identical, and different from the other G-quadruplex (Fig. S6†).

**Fig. 3:**
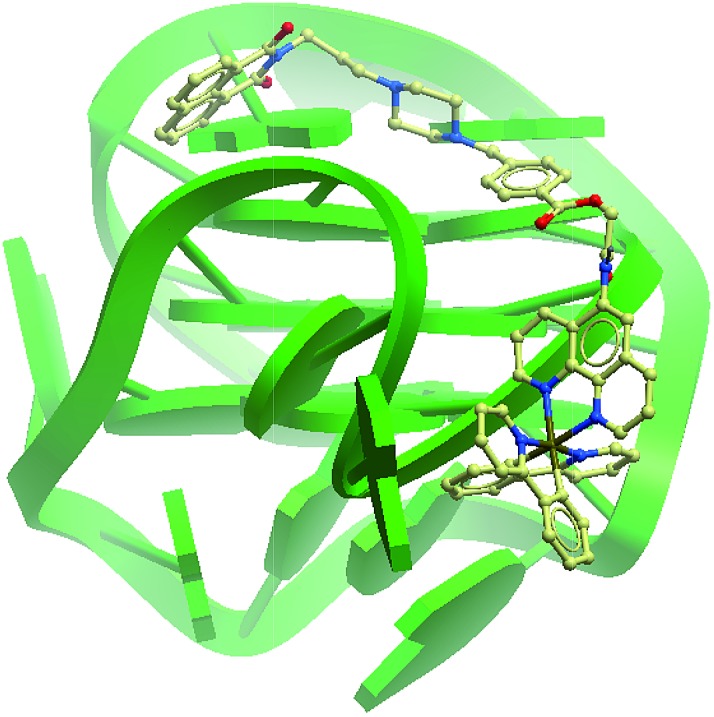
Side view of the interaction of **1** with the G-quadruplex structure in a hypothetical molecular model. The G-quadruplex is depicted as a ribbon representation (green), while **1** is depicted as a space-filling representation showing carbon (beige), oxygen (red) and nitrogen (blue).

The G-quadruplex binding of **1** was further examined utilizing the Molsoft ICM method (ICM-Pro 3.6-1d molecular docking software).^43^ Firstly, the geometry of **1** was optimized using density functional theory (DFT) calculations. We initially studied the binding of complex **1** to an intramolecular (3+1) G-quadruplex with a long central loop (PDB: ; 2LOD)^44^ to study the loop binding behaviour of **1**. In the low energy binding pose of **1** and G-quadruplex, **1** is predicted to interact with the A3–T4 loop as well as the G1–G3 and G7–G8 grooves (Fig. 3). While the binding unit **3** was also predicted to interact with the bases of G17 and C19 in the 3′-side loop. Since no salt-bridge interaction or hydrogen bonding was predicted in this model, we anticipate that the interaction between G-quadruplex and **1** is mainly attributed to the hydrophobic interaction between the groove and binding motif **3**. To further investigate the G-quadruplex-binding mode of **1**, we calculated the predicted binding pose of **1** towards ckit1 (PDB: ; 4WO3)^45^ and human telomeric G-quadruplex (PDB: ; 1KF1).^46^ The lowest-energy binding poses revealed that **1** binds to the loop region of both the ckit1 and human telomeric G-quadruplex structures (Fig. S7†). Complex **1** is predicted to interact with the T12–A13 loop as well as the G14–G16 groove of human telomeric G-quadruplex. Additionally, **1** was predicted to interact with C9 and A19 in the loop as well as the G6–G8 groove of the ckit1 G-quadruplex. The molecular docking results further validate the groove-binding behaviour of **1** that is conferred by the recognition unit **3**.

Interestingly, **1** showed a reduced luminescence enhancement for the AGR2 protein compared to the parent complex **2**. Complex **2** showed a *ca.* 1.6-fold enhancement in the presence of AGR2, while the binder linked **1** showed no significant enhancement (Fig. S1b†). This may be due to the larger three-dimensional nature of the conjugated complex **1**, which may reduce non-specific binding of the complex towards proteins.

Given the encouraging selectivity of complex **1** towards G-quadruplex DNA compared to other types of DNA or proteins, we employed **1** to construct a G-quadruplex-based AGR2 sensing platform to demonstrate the proof-of-concept application of a linked Ir(iii) complex for DNA-based sensing. The AGR2 sensing platform is depicted schematically in Fig. 1b. **Probe A** containing a G-quadruplex-forming sequence and the AGR2 aptamer sequence was partially hybridized to its antisense sequence (**Probe B**) to form a duplex substrate. The addition of AGR2 will cause the dissociation of the duplex substrate due to the strong binding of AGR2 to its aptamer sequence. The liberated G-quadruplex sequence will then fold into a G-quadruplex structure upon the addition of potassium ions, and will be recognized by **1** with a strong luminescence response. In this design, **Probe A**, which contains both the G-quadruplex and AGR2 aptamer sequences, was hybridized by the antisense DNA, **Probe B**. This **Probe B**, which hybridized only a part of the aptamer and G-quadruplex, resists the folding of G-quadruplex in the presence of potassium ions. Meanwhile the length of the **Probe B** strand is important for the sensitivity. An excessively long **Probe B** may cause the probe DNA duplex to become extraordinarily stable and resist the dissociation by aptamer-target binding, or prevent the G-quadruplex sequence from folding to the G-quadruplex after the probe DNA duplex binds to AGR2. Meanwhile, an excessively short **Probe B** may not fully prevent the folding of **Probe A**, resulting in a background signal increase. As a result, the shortest ckit1 G-quadruplex was selected for AGR2 sensing among the three highest fold enrichment G-quadruplexes.

To demonstrate the feasibility of our sensing platform, 80 nM AGR2 was introduced into a solution containing 0.5 μM hybridized duplex substrate. After incubation at 37 °C for 45 min, KCl was added to facilitate the formation of the G-quadruplex, followed by the addition of 0.5 μM **1**. Encouragingly, we observed that the system showed an obvious luminescence enhancement in the presence of AGR2 (Fig. S8†). To improve the performance of the sensing platform, we further optimized several experimental parameters that are relevant for this assay. We found that the relative luminescence enhancement of the system was highly dependent on the concentration of complex **1**, with a maximal response obtained using 0.5 μM complex. Furthermore, we also optimized the DNA and potassium concentration for better sensitivity (Fig. S9†).

**Fig. 4:**
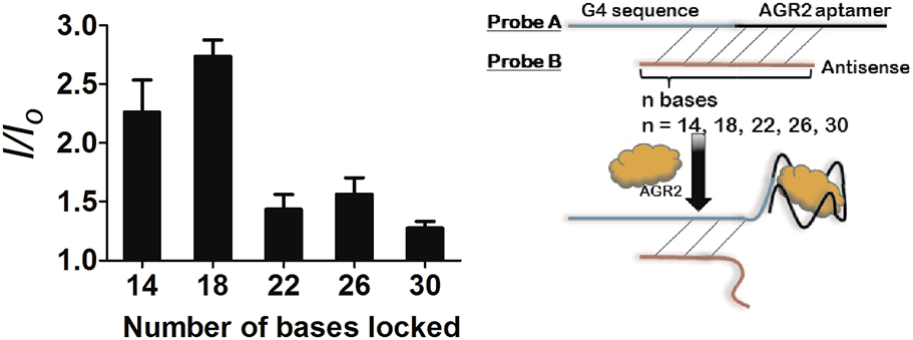
Luminescence enhancement of the system with different numbers of hybridized bases in the presence of 100 nM AGR2.

After optimization, the length of **Probe B** was also found to be important for the sensitivity of the assay. If **Probe B** is too long, the duplex substrate may be too stable and resist dissociation upon the addition of AGR2, thus impacting the detection limit. On the other hand, an excessively short **Probe B** sequence may permit partial dissociation of the duplex substrate even in the absence of AGR2, thus raising the background signal. We found that the fold enhancement of the system was at a maximum when the length of **Probe B** was 18 bases (Fig. 4).

After optimization of the experimental parameters, we investigated the luminescence response of the system to different concentrations of AGR2. Encouragingly, the luminescence intensity increased as more AGR2 was added (Fig. S10†). The limit of detection of the assay (path A) was 10 nM at a signal-to-noise ratio of 3, and a linear detection range of target AGR2 from 10 to 60 nM with a maximum 2.7-fold enhancement was observed.

If **1** was added to AGR2 only, no enhanced luminescence was observed (Fig. S11a†), indicating that **1** did not directly interact with AGR2. Thus, we envisage that the luminescence enhancement of **1** was due to the specific binding of AGR2 to its aptamer, which promotes the dissociation of the duplex substrate and the formation of the G-quadruplex motif in the presence of K^+^ ions that is recognized by **1**. To further verify the mechanism of this assay, we designed mutant probe DNA sequences that are unable to form a G-quadruplex structure and are also unable to bind AGR2, due to the lack of key guanine residues and specific mutations in the aptamer sequence. No significant change in the luminescence signal of **1** was displayed when mutant **Probe A** and **Probe B** were incubated with AGR2 (Fig. S11b†). Another control experiment using mutant **Probe A**_**mutant2**_ that bears an intact AGR2 aptamer segment but cannot form the ckit1 G-quadruplex was carried out. No significant enhancement was observed even upon the addition of 80 nM AGR2 (Fig. S11c†). This demonstrates that the signal enhancement originates from the formation of the ckit1 G-quadruplex, rather than the AGR2-aptamer complex. This result suggests that the formation of the ckit1 G-quadruplex motif was important for the luminescence enhancement of the system. Circular dichroism (CD) spectroscopy was performed to demonstrate the expected DNA conformational change in this assay. Firstly, the addition of AGR2 into buffer alone did not produce a significant change in the CD signal (data not shown). However, the addition of AGR2 to the duplex substrate induced the formation of a positive band at about 260 nm and a slight negative peak at about 235 nm, which are characteristic signals for parallel G-quadruplex DNA. Taken together, these data suggest that the luminescence enhancement of the system originated from the specific interaction of **1** with the G-quadruplex motif, which is in turn a consequence of the dissociation of the duplex DNA substrate caused by the specific binding of AGR2 (Fig. S12†).

**Fig. 5:**
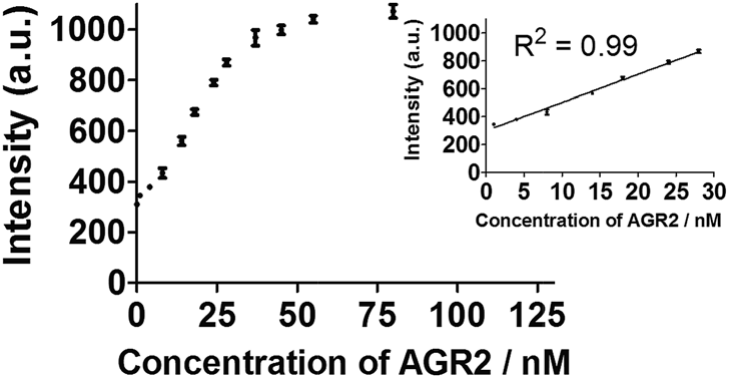
Linear plot of the change in luminescence intensity at *λ* = 585 nm *vs.* AGR2 concentration using the sensing mechanism path B.

To further improve the sensitivity of the AGR2 detection assay, we set out to reduce the background signal of the assay. A significant contribution to the background signal is due to the undesirable binding of **1** to ssDNA. Thus we introduced enzymes capable of DNA digestion. We employed exonuclease III (ExoIII) and exonuclease I (ExoI) to catalyze the digestion of mononucleotides from the 3′-hydroxyl end of dsDNA and ssDNA, respectively.^47^,^48^ The assay is performed in a similar fashion to that previously described (path A in Fig. 1), but with the additional presence of ExoIII and ExoI (path B). The antisense DNA released after the dissociation of the duplex substrate, as well as the intact duplex DNA, will be digested by ExoI and ExoIII, respectively. In contrast, the folded G-quadruplex structure and AGR2-bound aptamer sequence resist digestion by ExoIII and ExoI. As a consequence, the background signal caused by the undesirable binding of complex **1** to ssDNA and dsDNA will be eliminated. Using the modified method (path B), the maximum fold-enhancement was increased to 3.5-fold from 2.6-fold, while the detection limit for AGR2 was lowered to 1 nM with a linear range of detection from 1 to 28 nM (Fig. 5 and S13†).

**Fig. 6:**
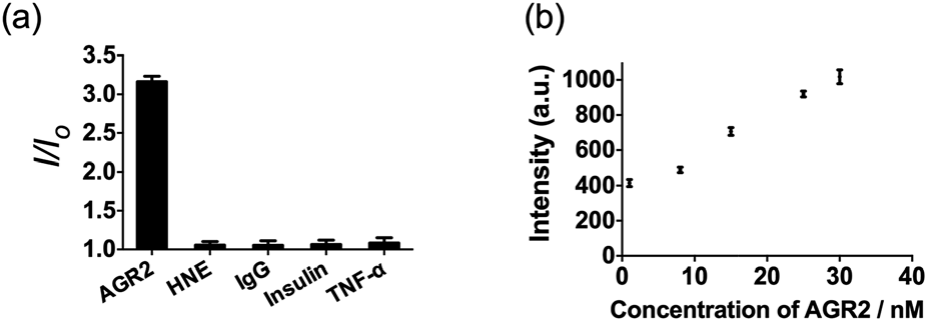
(a) Relative luminescence intensity of the system (path B) in the presence of 50 nM AGR2 or 250 nM other proteins. (b) Luminescence response of the system (path B) in the presence of increasing concentrations of AGR2 in 2.5% (*v/v*) FBS.

Additionally, to evaluate the selectivity of this assay, we investigated the response of the system to human neutrophil elastase (HNE), immunoglobulin G (IgG), insulin and tumor necrosis factor alpha (TNF-α). The AGR2 protein induced the highest luminescence enhancement of the system among the various substances under study (Fig. 6a), indicating the high selectivity of this aptamer-based assay for the detection of AGR2. We attribute the high selectivity of this assay to the high specificity of the AGR2 aptamer for its cognate target.

We next examined the suitability of the assay to detect AGR2 in a biological matrix. We performed the detection assay in a sensing system containing 2.5% (*v/v*) fetal bovine serum. We observed that the **1**/DNA system showed an enhanced luminescence intensity in diluted fetal bovine serum as the concentration of AGR2 was increased (Fig. 6b). This result demonstrates the possibility of using this approach for the quantification of AGR2 in serum for cancer diagnosis.

## Conclusions

In conclusion, we have successfully linked a known G-quadruplex groove binder, a benzo[*d*,*e*]isoquinoline motif, to an Ir(iii) complex, thus generating a highly selective G-quadruplex probe **1** which showed advantages of both parent complex **2** and groove binder **3**. Similar to **3**, the conjugated complex **1** exhibits a superior affinity and selectivity for G-quadruplex DNA over other conformations of DNA or proteins, with the fold enhancement ratio obviously improved compared with parent complex **2**. The molecular modelling revealed a groove-binding mode between complex **1** and G-quadruplex. Meanwhile it also possesses the prominent advantages of transition metal complex probes such as a large Stokes shift and long lifetime phosphorescence. We successfully employed time-resolved emission spectroscopy (TRES) to demonstrate the detectability of the long lifetime luminescence of **1** in strong fluorescence media. We then employed **1** to develop a G-quadruplex-based sensing system for the detection of AGR2, a potential serum biomarker for cancer, as a “proof-of-principle” concept. A detection limit of 1 nM for AGR2 was achieved using this label-free method with superior selectivity over a variety of other proteins, and the assay could function effectively for AGR2 detection in diluted fetal bovine serum. We anticipate that this conjugation method may be further employed for the development of G-quadruplex probes as well as for the detection of specific biomarkers associated with various human diseases.

## Experimental section

### Materials

Immunoglobulin G, insulin, coumarin, perylene, rhodamine B and other reagents, unless specified, were purchased from Sigma Aldrich (St. Louis, MO) and used as received. Iridium chloride hydrate (IrCl_3_·*x*H_2_O) was purchased from Precious Metals Online (Australia). All oligonucleotides were synthesized by Techdragon Inc. (Hong Kong, China). Fetal bovine serum 10270 (GIBCO®, origin: South America, EU approved origin) was purchased from Life Technologies (Grand Island, NY, USA). Human tumor necrosis factor-alpha was purchased from Sangon Biotech (Shanghai, China). Human neutrophil elastase was purchased from Innovative Research (Novi, MI, USA).

### General experimental

Mass spectrometry was performed at the Mass Spectroscopy Unit at the Department of Chemistry, Hong Kong Baptist University, Hong Kong (China). Deuterated solvents for NMR purposes were obtained from Armar and used as received. Circular dichroism (CD) spectra were collected on a JASCO-815 spectrometer. Steady state emission spectra were recorded on a QM-4 Photon Technology International instrument while time-resolved emission spectra were measured on a Horiba Fluorolog TCSPC spectrophotometer.

^1^H and ^13^C NMR spectra were recorded on a Bruker Avance 400 spectrometer operating at 400 MHz (^1^H) and 100 MHz (^13^C). ^1^H and ^13^C chemical shifts were referenced internally to a solvent shift (acetone-*d*_6_: ^1^H *δ* 2.05 and ^13^C *δ* 29.8; CD_3_Cl: ^1^H *δ* 7.26 and ^13^C *δ* 76.8). Chemical shifts (*δ*) are quoted in ppm, the downfield direction being defined as positive. Uncertainties in chemical shifts are typically ±0.01 ppm for ^1^H and ±0.05 for ^13^C. Coupling constants are typically ± 0.1 Hz for ^1^H–^1^H and ±0.5 Hz for ^1^H–^13^C couplings. The following abbreviations are used for convenience in reporting the multiplicity of NMR resonances: s, singlet; d, doublet; t, triplet; q, quartet; m, multiplet; br, broad. All NMR data was acquired and processed using standard Bruker software (Topspin).

### FRET melting assay

The ability of **1** to stabilize G-quadruplex DNA was investigated using a fluorescence resonance energy transfer (FRET) melting assay. The labelled G-quadruplex-forming oligonucleotide F21T (5′-FAM-d(G_3_[T_2_AG_3_]_3_)-TAMRA-3′; donor fluorophore FAM: 6-carboxyfluorescein; acceptor fluorophore TAMRA: 6-carboxytetramethylrhodamine) was diluted to 200 nM in potassium cacodylate buffer (100 mM KCl, pH 7.0), and then heated to 95 °C in the presence of the indicated concentrations of **1**. The labeled duplex-forming oligonucleotide F10T (5′-FAM-dTATAGCTA-HEG-TATAGCTATAT-TAMRA-3′) (HEG linker: [(–CH_2_–CH_2_–O–)_6_]) was treated in the same manner, except that the buffer was changed to 10 mM lithium cacodylate (pH 7.4). Fluorescence readings were taken at intervals of 0.5 °C over the range of 25 to 95 °C.

## Supplementary Material

Supplementary information
