# Supplementary material for: Conjugating a groove-binding motif to an Ir(iii) complex for the enhancement of G-quadruplex probe behavior
Source: Chem Sci. 2016 Jan 28;7(4):2516–23. doi: 10.1039/c6sc00001k (PMC5477052; doi:10.1039/c6sc00001k)
Supplement: Supplementary file 1 [file SC-007-C6SC00001K-s001.pdf]

Electronic Supplementary Material (ESI) for Chemical Science.
This journal is © The Royal Society of Chemistry 2016

Electronic Supporting Information

Conjugating a groove-binding motif to Ir(III) complex for the enhancement of G-quadruplex probe behavior

Modi Wang,‡[a] Zhifeng Mao,‡[a] Tian-Shu Kang,[b] Chun-Yuen Wong,[c] Jean-Louis Mergny,*[de] Chung-Hang Leung*[b] and Dik-Lung Ma*[a]

[a] Department of Chemistry, Hong Kong Baptist University, Kowloon Tong, Hong Kong, China. Email: edmondma@hkbu.edu.hk

[b]State Key Laboratory of Quality Research in Chinese Medicine, Institute of Chinese Medical Sciences, University of Macau, Macao, China. Email: duncanleung@umac.mo

[c]Department of Biology and Chemistry, City University of Hong Kong, Kowloon Tong, Hong Kong, China

[d]University of Bordeaux, ARNA laboratory, Bordeaux, France. Email: jean-louis.mergny@inserm.fr

[e]INSERM, U869, IECB, Pessac, France.

‡ These authors contributed equally to this work.

Experimental section

Photophysical measurement. Emission spectra and lifetime measurements for complexes were performed on a PTI TimeMaster C720 Spectrometer (Nitrogen laser: pulse output 337 nm) fitted with a 380 nm filter. Error limits were estimated: λ (±1 nm); τ (±10%); φ (±10%). All solvents used for the lifetime measurements were degassed using three cycles of freeze-vac-thaw.

Luminescence quantum yields were determined using the method of Demas and Crosby[1] $[Ru(bpy)_3][PF_6]_2$ in degassed acetonitrile as a standard reference solution (Φ_r = 0.062) and calculated according to the following equation:

$$\Phi_s = \Phi_r(B_r/B_s)(n_s/n_r)^2(D_s/D_r)$$

where the subscripts s and r refer to sample and reference standard solution respectively, n is the refractive index of the solvents, D is the integrated intensity, and

Φ is the luminescence quantum yield. The quantity B was calculated by $B = 1 - 10^{-AL}$, where A is the absorbance at the excitation wavelength and L is the optical path length.

Theoretical Calculations

The molecular structure of **1** were optimized at the density functional theory (DFT) level using Becke's three-parameter hybrid functional with the Lee-Yang-Parr correlation functional (B3LYP).[2, 3] The LANL2DZ pseudopotential basis set was employed for the Ir or Rh atom; the 6-31G* basis set was employed for C, H, and N atoms.[4] Tight SCF convergence (10–8 au) was used for all calculations. All the calculations were performed using the Gaussian 09 program package (revision B.01).

Molecular Modeling

Molecular docking was performed by using the ICM-Pro 3.6-1d program (Molsoft).[5, 6] According to the ICM method, the molecular system was described by using internal coordinates as variables. Energy calculations were based on the ECEPP/3 force field with a distance-dependent dielectric constant. The biased probability Monte Carlo (BPMC) minimization procedure was used for global energy optimization. The BPMC global-energy-optimization method consists of 1) a random conformation change of the free variables according to a predefined continuous probability distribution; 2) local-energy minimization of analytical differentiable terms; 3) calculation of the complete energy including nondifferentiable terms such as entropy and solvation energy; 4) acceptance or rejection of the total energy based on the Metropolis criterion and return to step (1). The binding between **1** and DNA was evaluated by binding energy, including grid energy, continuum electrostatic, and entropy terms. The initial model of loop isomer was built from the X-ray crystal structures of G quadruplex, according to a previously reported procedure.[7, 8] Briefly, the structure of G quadruplex was imported into Insight II package (Accelrys Inc., San Diego, CA), and necessary modifications were carried out including replacements and deletions of bases. Missing loop nucleotides were added using single-strand B-DNA geometry using the Biopolymer module. Potassium ions were placed between the G-tetrad planes to stabilize the tetrad structure. The initial models were then immersed in a box of TIP3P water molecules, and an appropriate number of sodium ions was added to neutralize the negative charge of the phosphate backbone. The molecular dynamics simulations were carried out in NAMD with VMD monitoring

the process. The CHARMM force field parameter was assigned to every atom, and the Particle Mesh Ewald electrostatics was used to compute long-range electrostatic interactions. Hydrogen atoms were added and minimized by 3000 steps of conjugate gradient minimization. After 4000 steps of conjugate gradient minimization, two stages of molecular dynamics simulations were carried out at 300 K. In the first stage, only the loop area atoms were allowed to move, and this process involved a 20 ps equilibration and 100 ps simulations. The second stage involved unrestrained molecular dynamics simulations with 20 ps equilibration and 100 ps simulations at 300 K. Trajectories were recorded every 0.1 ps, and the most stable structure was extracted and further refined by 2500 steps of conjugate gradient minimization. In the docking analysis, the binding site was assigned to the groove regions of the DNA molecule. The ICM docking was performed to find the most favorable orientation. The resulting trajectories of the complex between **1** and G-quadruplex DNA were energy minimized, and the interaction energies were computed.

Synthesis

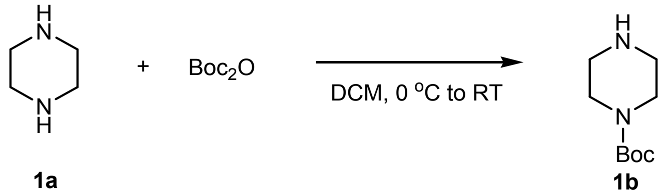

Compound **1b** was synthesized using a modified literature method.[9] A solution of piperazine **1a** (2.58 g, 30 mmol) in dichloromethane (75 mL) was cooled to 0 °C on an ice bath. A solution of di-*tert*-butyldicarbonate (3.27 g, 15 mmol) in dichloromethane (30 mL) was added dropwise over 10 minutes with vigorous stirring, forming a white precipitate. The solution was removed from the ice bath and stirred under ambient conditions for 3 h. The mixture was filtered and the solvent was removed by rotary evaporation to give a clear oil. Water (30 mL) was added, forming a white precipitate. The mixture was filtered, saturated with potassium carbonate, and extracted with diethyl ether (3 x 125 mL). The organic layers were combined, dried over sodium sulfate, and evaporated to give the product as hygroscopic white crystals. Yield: 76%.

^{1}H-NMR (400 MHz; $CDCl_3$): δ 3.34 (t, *J* = 5.2 Hz, 4H), 2.76 (t, *J* = 5.2 Hz, 4H), 1.74 (s, 1H), 1.42 (s, 9H). ^{13}C-NMR (400 MHz; $CDCl_3$): δ 154.7, 79.5, 45.8, 44.4, 28.4.

HRMS: Calcd. for $C_9H_{19}N_2O_2$: *m/z* = 187.1447. Found: *m/z* = 187.1444. [M+H].

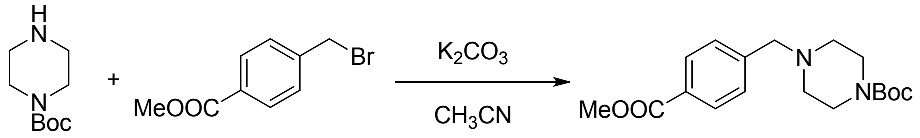

1b **1c**

The solution of methyl 4-(bromomethyl)benzoate (1.55 g, 6.77 mmol), K_2CO_3 (1.77 g, 12.8 mmol) in CH_3CN was cooled to 0 °C, then tert-butyl 1-piperazinecarboxylate **1b** (1.4 g, 7.53 mmol) was added. The mixture was stirred overnight at room temperature. The mixture was filtered through a Celite pad and evaporated to dryness to give the product as a yellow oil, which was purified by silica gel column chromatography (eluent, ethyl acetate) to afford the desired **1c**. Yield: 91%.

^{1}H-NMR (400 MHz; $CDCl_3$): δ 7.97 (dd, *J* = 6.8, 1.6 Hz, 2H), 7.39 (d, *J* = 8.4 Hz, 2H), 3.90 (s, 3H), 3.54 (s, 2H), 3.42 (t, *J* = 5.2 Hz, 4H), 2.37 (t, *J* = 4.4 Hz, 4H), 1.44 (s, 9H). ^{13}C-NMR (400 MHz; $CDCl_3$): δ 166.9, 154.7, 143.4, 129.6, 129.0, 128.9, 79.6, 62.6, 52.9, 52.0, 43.2, 28.4. HRMS: Calcd. for $C_{18}H_{27}N_2O_4$: *m/z* = 335.1965. Found: *m/z* = 335.1945. [M+H].

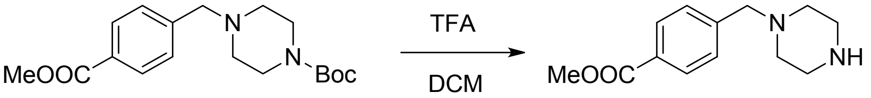

1c **1d**

Tert-butyl 4-(4-(methoxycarbonyl)benzyl)piperazine-l-carboxylate **1c** (1.5 g, 4.49 mmol) was dissolved in a mixture of trifluoroacetic acid (4.0 mL) and dichloromethane (13.5 mL). The solution was stirred at room temperature overnight, and then was evaporated to dryness in vacuum to give the product as colourless oil. Yield: 95%.

^{1}H-NMR (400 MHz; DMSO-d_6): δ 7.95 (dd, *J* = 6.4, 1.6 Hz, 2H), 7.48 (d, *J* = 8.4 Hz, 2H), 3.86 (s, 3H), 3.63 (s, 2H), 3.10 (t, *J* = 4.8 Hz, 4H), 2.58 (t, *J* = 4.8 Hz, 4H). ^{13}C-NMR (400 MHz; DMSO-d_6): δ 166.1, 143.1, 129.2, 129.0, 128.5, 60.9, 52.1, 49.1, 42.9. HRMS: Calcd. for $C_{13}H_{19}N_2O_2$: *m/z* = 235.1447. Found: *m/z* = 235.1460. [M+H].

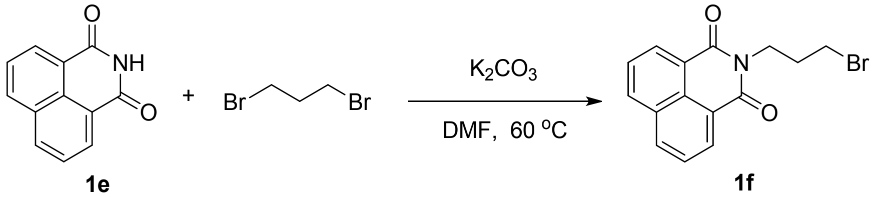

1e **1f**

Compound **1f** was synthesized using a modified literature method.[10] To a solution of

compound **1e** (1.18 g, 6.0 mmol) in DMF (40 mL) was added potassium carbonate (3.32 g, 24.0 mmol). After the reaction was stirred at 40 °C for 1 h, 1,3-dibromopropane (4.85 g, 24.0 mmol) was added, and the resulting mixture was stirred overnight at 50 °C. The mixture was cooled to room temperature. The mixture was extracted with EA and washed with water. Subsequently, the organic extracts were collected, dried over anhydrous sodium sulfate and concentrated under reduced pressure to give the crude material, which was purified by silica gel column chromatography (eluent, dichloromethane/hexane, 1:2, *v/v*) to afford the desired naphthalimide bromide **1f**. Yield: 76%. ^{1}H-NMR (400 MHz; $CDCl_3$): δ 8.59 (dd, J = 7.2, 1.2 Hz, 2H), 8.21 (dd, J = 8.8, 1.2 Hz, 2H), 7.77-7.73 (m, 2H), 4.33 (t, J = 6.8 Hz, 2H), 3.50 (t, J = 7.2 Hz, 2H), 2.37-2.30 (m, 2H). ^{13}C-NMR (400 MHz; $CDCl_3$): δ 164.2, 134.0, 131.6, 131.3, 128.1, 126.9, 122.5, 39.3, 31.4, 30.5. HRMS: Calcd. for $C_{15}H_{13}BrNO_2$: *m/z* = 318.0124. Found: *m/z* = 318.0137. [M+H].

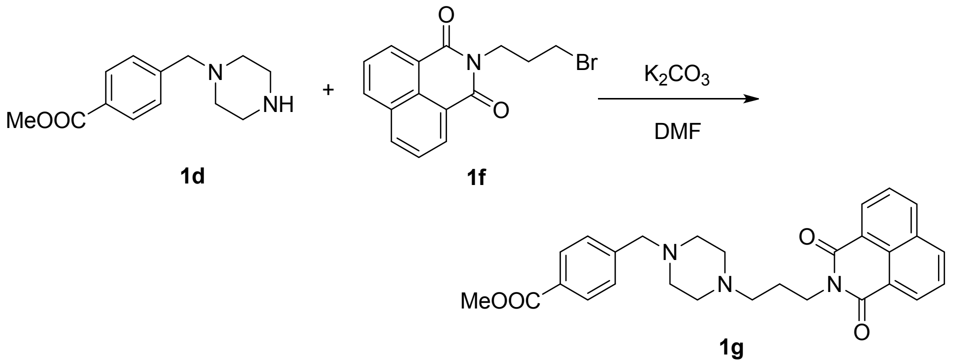

Compound **1g** was synthesized using a modified literature method.[11] A suspension of the compound **1d** (0.70 g, 3.0 mmol) in DMF (18 mL) and K_2CO_3 (829 mg, 6 mmol) was heated at 80 °C for 10 min. Compound **1f** (881 mg, 3.3 mmol) were then added and the reaction was stirred at the same temperature for 6 h. After cooling at room temperature, the solvent was evaporated and the residue partitioned between water (50 mL) and ethyl acetate (50 mL). The aqueous phase was further extracted with ethyl acetate (3 × 30 mL) and the combined organic layers were dried over Na_2SO_4, evaporated to dryness and then was purified by silica gel column chromatography (eluent, ethyl acetate/dichloromethane, 1:1, *v/v*) to afford the desired product **1g**. Yield: 72%.

^{1}H-NMR (400 MHz; $CDCl_3$): δ 8.58 (dd, J = 7.2, 0.8 Hz, 2H), 8.20 (dd, J = 8.4, 0.8 Hz, 2H), 7.95 (d, J = 8.4 Hz, 2H), 7.75 (m, 2H), 7.34 (d, J = 8.4 Hz, 2H), 4.24 (t, J =

7.2 Hz, 2H), 3.90 (s, 3H), 3.44 (s, 2H), 2.51 (t, J = 7.2 Hz, 2H), 2.46-2.36 (m, 6H), 1.97-1.90 (m, 2H), 1.79 (s, 2H). ^{13}C-NMR (400 MHz; $CDCl_3$): δ 167.0, 164.2, 143.8, 133.8, 131.6, 131.1, 129.5, 128.9, 128.9, 128.2, 126.9, 122.8, 62.6, 56.1, 53.1, 53.0, 52.0, 38.9, 25.0. HRMS: Calcd. for $C_{28}H_{30}N_3O_4$: m/z = 472.2231. Found: m/z = 472.2254. [M+H].

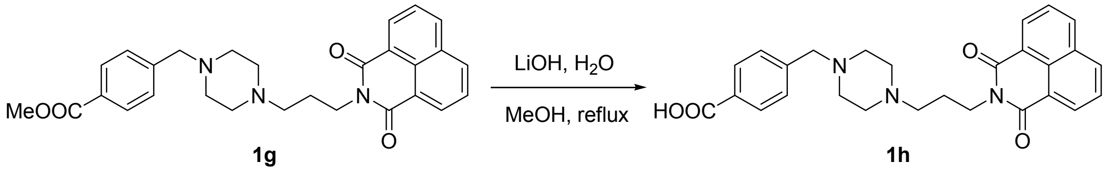

A solution of lithium hydroxide (73.5 mg, 1.75 mmol) in H_2O (2 mL) was added to a solution of Intermediate **1g** (330 mg, 0.70 mmol) in methanol (6 mL) and the mixture was heated at reflux for 2 h. The mixture was concentrated under reduced pressure. 30 mL water was added to the mixture and then the mixture was acidified with aqueous HCl until a white solid formed. Because compound **1h** has poor solubility, it was used without further purification.

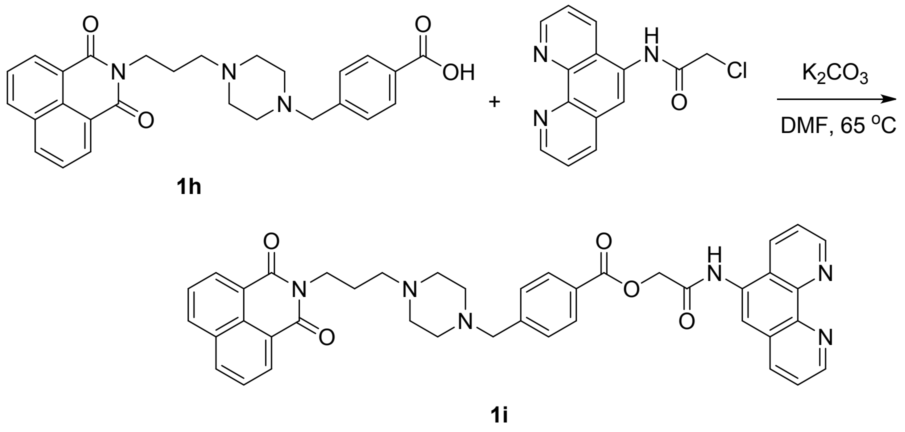

To a solution of compound **1h** (0.318 g, 0.696 mmol) and potassium carbonate (0.132 g, 0.949 mmol) in N, N-dimethylformamide (10 mL), was added 2-chloro-N-(1,10-phenanthrolin-5-yl)acetamide (0.171 g, 0.633 mmol). After stirring at 65 °C overnight, the reaction mixture was diluted with ethyl acetate (25 mL), washed with water and brine. The organic phase was dried over Na_2SO_4, filtered and isolated by silica gel flash chromatography (eluent, methanol/dichloromethane, 1:10, *v/v*) to obtain the product **1i**. Yield: 75%.

^{1}H-NMR (400 MHz; $CDCl_3$): δ 9.36 (s, 1H), 8.98 (t, J = 4.4 Hz, 2H), 8.49 (dd, J = 7.2, 0.8 Hz, 2H), 8.32 (d, J = 8.4 Hz, 1H), 8.13 (d, J = 7.6 Hz, 2H), 8.08-7.98 (m, 4H), 7.67 (t, J = 7.6 Hz, 2H), 7.50-7.42 (m, 2H), 7.32 (d, J = 8.4 Hz, 2H), 5.09 (s, 2H),

4.17 (t, J = 7.2 Hz, 2H), 3.41 (s, 2H), 2.49-2.34 (m, 10H), 1.92-1.85 (m, 2H). ^{13}C-NMR (400 MHz; $CDCl_3$): δ 166.9, 165.7, 164.1, 149.9, 149.7, 146.1, 144.7, 144.3, 135.8, 133.8, 131.4, 131.0, 130.2, 129.9, 129.7, 129.1, 128.0, 127.9, 127.4, 126.8, 124.2, 123.3, 122.7, 122.5, 120.3, 63.8, 62.3, 55.8, 52.8, 38.7, 29.6, 24.8. HRMS: Calcd. for $C_{41}H_{36}N_6O_5$: m/z = 693.2820. Found: m/z = 693.2824. [M+H].

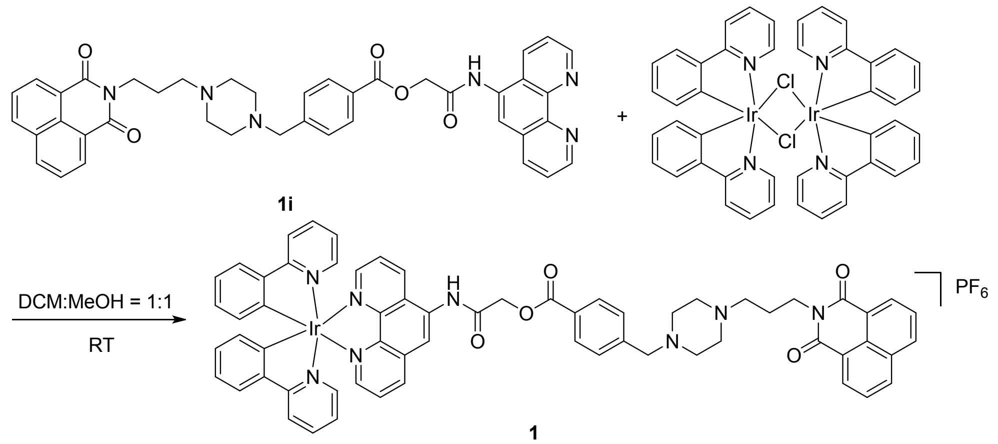

Complex **1** was synthesized using a modified literature method.[12] A solution of ligand **1i** (21.3 mg, 0.031 mmol) and the dichloro-bridged $[Ir(ppy)_2Cl]_2$ (15 mg, 0.014 mmol) in dichloromethane (3 mL) and methanol (3 mL) was stirred at room temperature overnight. After the reaction completed, an excess of solid NH_4PF_6 was added and stirred for another 0.5 h at room temperature. The solvent was removed under reduced pressure and the residue was purified by silica gel column chromatography (eluent, methanol/dichloromethane, 1:10, *v/v*) to yield **1** as an orange powder. Yield: 70%.

^{1}H-NMR (400 MHz; Acetone-d_6): δ 10.12 (s, 1H), 9.05 (dd, J = 8.4, 0.8 Hz, 1H), 8.82 (dd, J = 8.0, 1.2 Hz, 1H), 8.67 (s, 1H), 8.54 (dd, J = 7.2, 1.2 Hz, 2H), 8.47-8.42 (m, 3H), 8.35 (dd, J = 4.8, 1.2 Hz, 1H), 8.22 (d, J = 8.0 Hz, 2H), 8.10-8.06 (m, 3H), 8.01 (dd, J = 8.4, 5.2 Hz, 1H), 7.93-7.85 (m, 6H), 7.69-7.67 (m, 2H), 7.45 (d, J = 8.0 Hz, 2H), 7.09-7.04 (m, 2H), 6.99-6.93 (m, 4H), 6.45-6.42 (m, 2H), 5.22 (dd, J = 16.8, 15.2 Hz, 2H), 4.22 (t, J = 7.2 Hz, 2H, 3.46 (s, 2H), 2.58-2.28 (m, 10H), 1.97-1.90 (m, 2H). ^{13}C-NMR (400 MHz; Acetone-d_6): δ 205.3, 167.8, 167.7, 167.2, 165.7, 163.8, 151.4, 150.5, 150.1, 149.7, 149.5, 147.4, 145.0, 144.9, 144.3, 144.2, 138.6, 138.3, 134.2, 134.0, 133.7, 131.9, 131.8, 131.7, 131.2, 130.6, 130.4, 130.3, 129.7, 129.0, 128.2, 128.0, 127.8, 127.2, 127.0, 126.6, 124.9, 123.5, 123.4, 122.9, 122.6, 120.8, 119.8, 63.4, 61.9, 55.9, 52.9, 52.6, 38.4, 24.4. HRMS: Calcd. for $C_{63}H_{52}IrN_8O_5$[M–PF6]+: 1193.3690 Found: 1193.3677. Anal. ($C_{63}H_{52}IrN_8O_5PF_6 \cdot 3.5H_2O$) C, H, N:

calcd 54, 4.24, 8; found: 53.67, 4, 8.17.

Preparation of oligonucleotides

The DNA was prepared as previously described.[13] Before analysis, oligonucleotides were heated at 95 °C for 5 min, then diluted into 100 mM Tris-HCl, pH 7.2, 50 mM KCl and cooled to room temperature.

Luminescence response of Ir(III) complexes towards different forms of DNA

The assay was performed as previously described.[13] Oligonucleotides and Ir(III) complex were mixed at 5 μM and 1 μM final concentrations, respectively. Samples were incubated in 50 mM Tris-HCl, pH 7.2, and 50 mM KCl while the final volume is 500 μL.

Fold change I_{DNA}/I_0 is defined as luminescence intensity at the maximum wavelength of complex in the presence of DNA (I_{DNA}) divided by the luminescence intensity at the maximum wavelength of complex alone (I_0). The luminescence selectivity ratio of complexes **1**–**2** (Fig. S1a) equal to the fold change of complexes **1**–**2** towards G-quadruplex ($I_{G\text{-}quadruplex}/I_0$) divided by the fold change of complexes **1**–**2** towards ssDNA (I_{ssDNA}/I_0) or dsDNA (I_{dsDNA}/I_0) which is equal to $I_{G\text{-}quadruplex}/I_{ssDNA}$ or $I_{G\text{-}quadruplex}/I_{dsDNA}$ respectively.

Circular dichroism (CD) measurement

CD spectra was recorded on a JASCO-815 spectropolarimeter using 1 cm path length quartz cuvettes. Spectra was collected between 220 nm and 335 nm, using 2 cm bandwidth, 100 nm min^{-1} scan speed and five scans. The data were baseline corrected using CD spectra of buffer alone.

Detection of AGR2 in aqueous solution

The random-coil oligonucleotides Probe A (100 μM) and Probe B (100 μM) were mixed in Tris buffer (20 mM, pH 7.0). The solution was heated to 95 °C for 10 min, cooled to room temperature at 0.1 °C/s, and further incubated at room temperature for 1 h to ensure formation of the duplex substrate. The annealed product was stored at –20 °C before use. 5 μL of 50 μM duplex DNA (Probe A and B) was added into 40 μL of Tris buffered solution (20 mM Tris-HCl, pH 7.0) with the indicated concentrations of AGR2. The mixture was then incubated at 37 °C for 45 min. For path A, the

mixture was subsequently diluted using Tris buffer (20 mM Tris, 50 mM KCl, pH 7.0) to a final volume of 499 μL. Finally, 1 μL of 0.5 mM of complex **1** was added to the mixture and the mixture was incubated for 5 min before measurement. Emission spectra were recorded by QM-4 Photon Technology International in the range of 500−750 nm using an excitation wavelength of 330 nm.

For path B, after the incubation of the mixture at 37 °C, ExoIII and ExoI were added into the solution at the concentration of 20 U/mL. And the mixture was incubated at 37 °C for 30 min, followed by the enzyme inactivation by 20 mM EDTA. The mixture was cooled down and subsequently diluted using Tris buffer (20 mM Tris, 50 mM KCl, pH 7.0) to a final volume of 499 μL. Finally, 1 μL of 0.5 mM of complex **1** was added to the mixture and the mixture was incubated for 5 min before measurement. Emission spectra were recorded by QM-4 Photon Technology International in the range of 500−750 nm using an excitation wavelength of 330 nm.

Time-resolved emission spectra (TRES) measurement

20 μL of 1 mM competitive organic dye was added into 20 μM complex **1** in 2 mL ACN. Steady state emission spectra of mixture were recorded by QM-4 Photon Technology International while TRES was measured by a Horiba Fluorolog TCSPC spectrophotometer.

Table S1. DNA sequences used in this project:

	Sequence
Probe A	$5'\text{-}CG_3TG_3AGT_2GTG_9TG_3AG_3T_2G_3AG_3CGCTG_3AG_2AG_3\text{-}3'$
Probe B	$5'\text{-}CTC_3A_2C_3TC_3AC_3\text{-}3'$
ssDNA	$5'\text{-}CTCAT_4C_2ATACAT_2A_3GATAGTCAT\text{-}3'$
dsDNA	$5'\text{-}A_2G_2T_2AGCGT_2AG_2AT_2ACGGCAGA_2G_2ATA_2C_2GTA_2TC_2TA_2CGCTA_2C_2T_2\text{-}3'$
c-*kit87up*	$5'\text{-}AG_3AG_3CGCTG_3AG_2AG_3\text{-}3'$
c-*kit1*	$5'\text{-}G_3AG_3CGCTG_3AG_2AG_3\text{-}3'$
Pu27	$5'\text{-}TG_4AG_3TG_4AG_3TG_4A_2G_2\text{-}3'$
Probe A_{mutant}	$5'\text{-}C\underline{T_3}T\underline{T_3}A\underline{A}T_2G\underline{AT_4}G_2\underline{T_3}T\underline{AT_2AT_2A}T_2G\underline{A_2T}G\underline{T_3A}CT\underline{T_3}AG_2A\underline{AT_2}\text{-}3'$
Probe B_{mutant}	$5'\text{-}C\underline{AT_2}CA_2\underline{TA_2\ TA_2T}AA_3\text{-}3'$
Probe $A_{mutant2}$	$5'\text{-}CG_3TG_3AGT_2GTG_9TG_3AG_3T_2G\underline{A_2T}G\underline{T_3A}CT\underline{T_3}AG_2A\underline{AT_2}\text{-}3'$
Probe $B_{mutant2}$	$5'\text{-}CAT_2CA_2C_3TC_3AC_3\text{-}3'$
F10T	5′-FAM-TATAGCTA-HEG-TATAGCTATAT-TAMRA-3′
F21T	$5'\text{-}FAM\text{-}(G_3[T_2AG_3]_3)\text{-}TAMRA\text{-}3'$

Table S2. Photophysical properties of **1**.

Complex	Quantum yield	λ_{em}/ nm	Lifetime/ µs	UV/vis absorption λ_{abs} / nm (ε/ dm^3 mol^{-1} cm^{-1})
**1**	0.0878	590	4.828	235 (1.13×10^5)

Fig. S1 (a) Diagrammatic bar array representation of the luminescence enhancement selectivity ratio of complexes **1** and **2** upon the addition of *c-kit87up*, *c-kit1* and *Pu27* G-quadruplex over ssDNA or dsDNA. (b) Diagrammatic bar array representation of the luminescence enrichment of **1** and **2** for AGR2 protein.

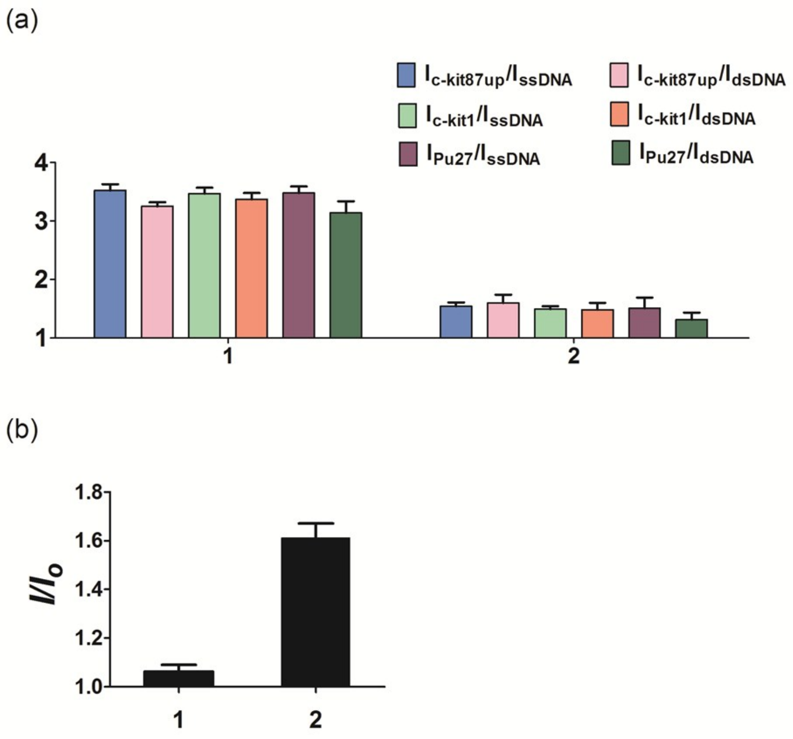

Fig. S2 (a) Melting profile of F21T G-quadruplex DNA (0.2 μM) in the absence and presence of **2** (5 μM). (b) Melting profile of F10T dsDNA (0.2 μM) in the absence and presence of **2** (5 μM).

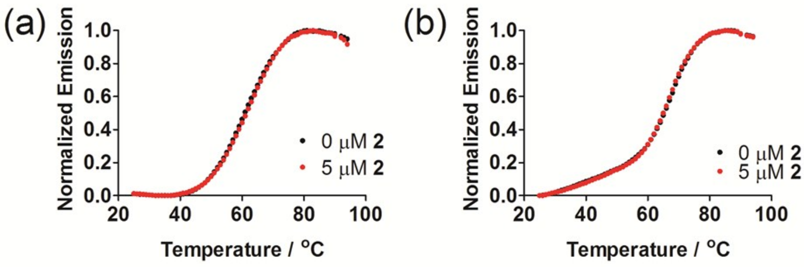

Fig. S3 (a) Melting profile of F21T G-quadruplex DNA (0.2 μM) in the absence and presence of **3** (5 μM). (b) Melting profile of F10T dsDNA (0.2 μM) in the absence and presence of **3** (5 μM).

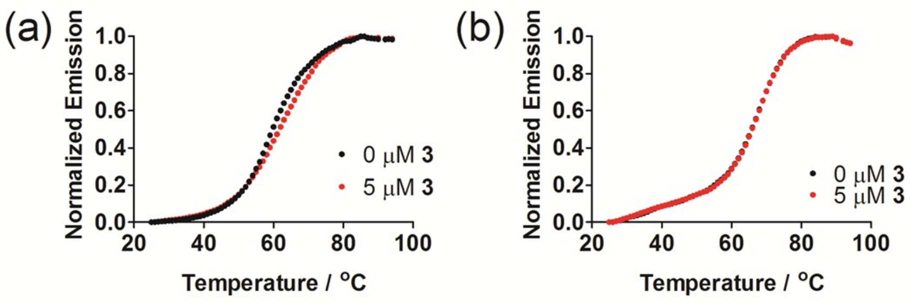

Fig. S4 (a) Melting profile of F21T G-quadruplex DNA (0.2 μM) in the absence and presence of **1** (5 μM). (b) Melting profile of F10T dsDNA (0.2 μM) in the absence and presence of **1** (5 μM). (c) Melting profile of F21T (0.2 μM) in the absence and presence of **1** (5 μM) and ds26 (10 μM) or ssDNA (10 μM).

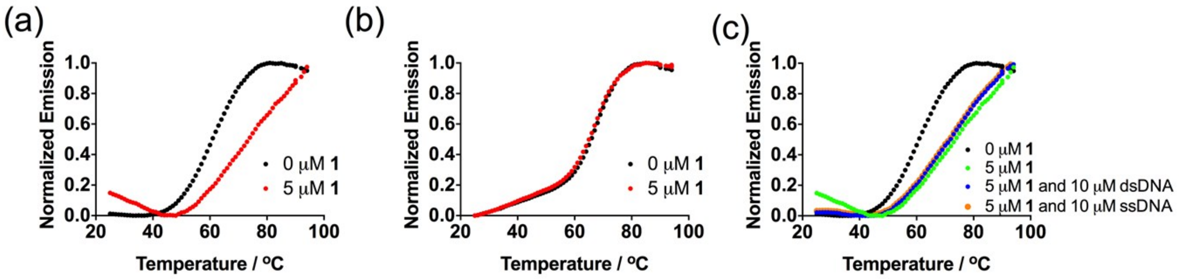

Fig. S5 Luminescence enhancement of complex **1** is shown as a function of loop size of 3′-side loop.

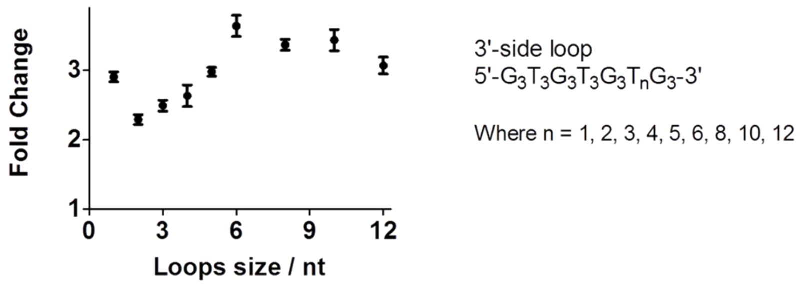

Fig. S6 Luminescence enhancement of complex **1** in the presence of TG_4T G-quadruplex (5′-TG_4T-3′) and *ckit1* G-quadruplex (5′-$G_3AG_3CGCTG_3AG_2AG_3$-3′).

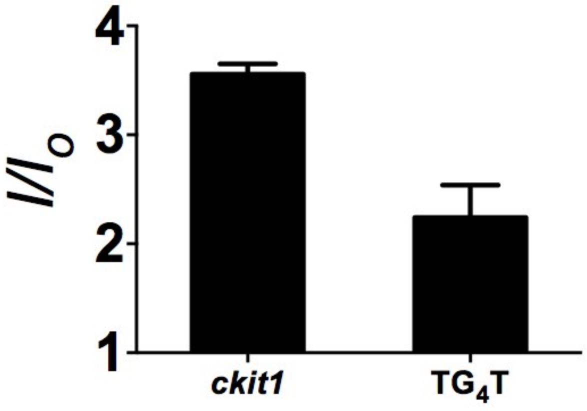

Fig. S7 Side view of the interactions of **1** with (a) the human telomeric G-quadruplex (PDB: 1KF1) and (b) the *ckit1* G-quadruplex (PDB: 4WO3) according to molecular modeling. The G-quadruplex is depicted as a ribbon representation (green), while **1** is depicted as a space-filling representation showing carbon (beige), oxygen (red) and nitrogen (blue).

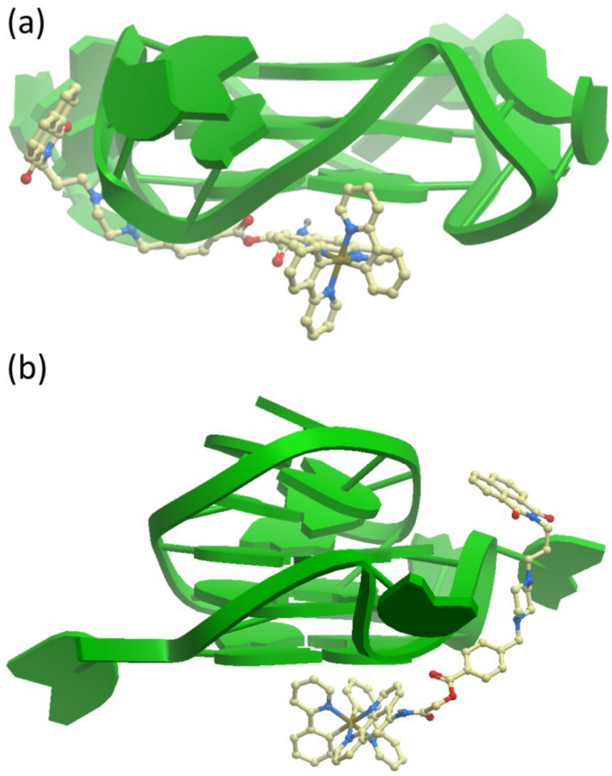

Fig. S8 Emission spectra of the system ([**1**] = 0.5 μM, [probe DNA duplex] = 0.5 μM, [K^+] = 50 mM) in the presence or absence of AGR2 (80 nM).

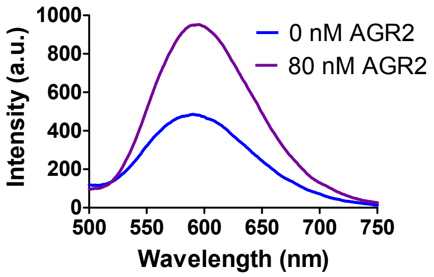

Fig. S9 (a) Relative luminescence intensity of the system with different concentrations of **1**. (b) Relative luminescence intensity of the system with different concentrations of probe DNA duplex. (c) Relative luminescence intensity with different concentrations of KCl. Unless otherwise stated, the concentration of complex **1** was 0.5 μM and the concentration of probe DNA duplex was 0.5 μM.

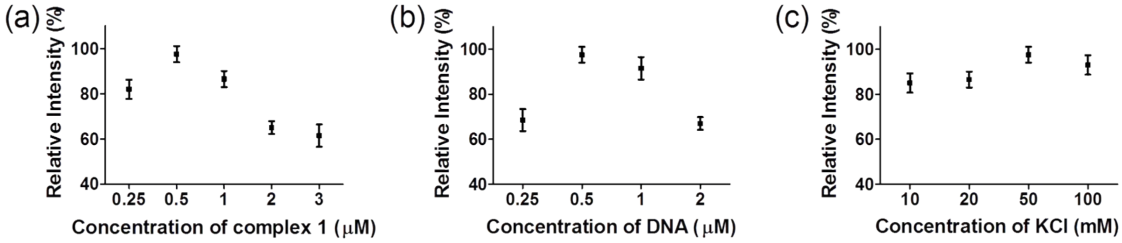

Fig. S10 (a) Emission spectrum of the system in the presence of increasing concentrations of AGR2 using the sensing mechanism path A. (b) Linear plot of the change in luminescence intensity at λ = 585 nm vs. AGR2 concentration.

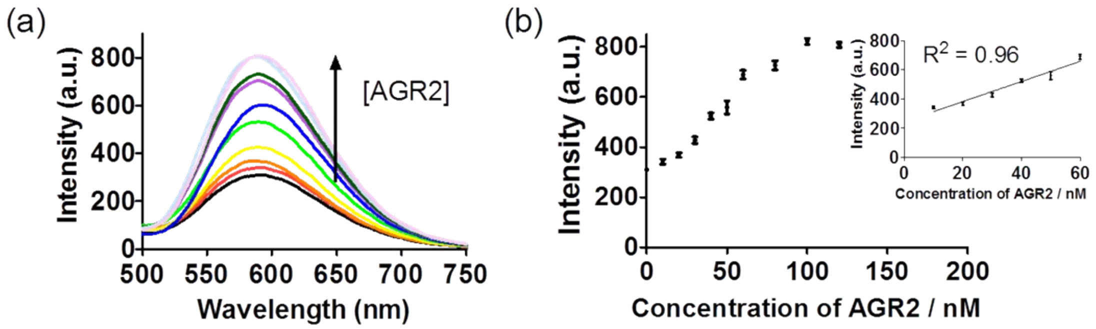

Fig. S11 (a) Luminescence enhancement of the system in response to AGR2 (80 nM) in the presence or absence of probe DNA duplex (0.5 μM). Relative luminescence response of the system using (b) wild-type and probe A_{mutant} or (c) wild-type and probe $A_{mutant2}$.

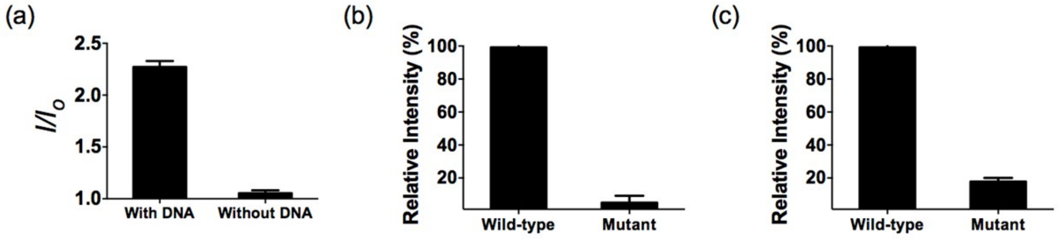

Fig. S12 CD spectrum of the sensing system (probe DNA = 2 μM) in the absence and presence of 100 nM AGR2.

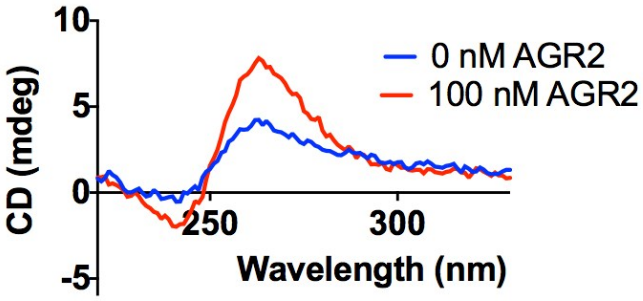

Fig. S13 Emission spectrum of the system in the presence of increasing concentrations of AGR2 using the sensing mechanism path B.

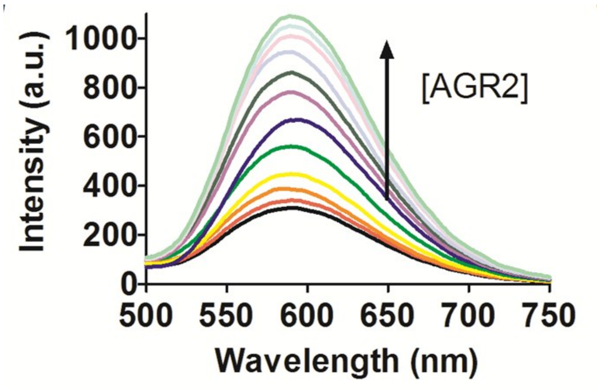

References

1. G. A. Crosby and J. N. Demas, *J. Phy. Chem.*, 1971, **75**, 991-1024.
2. A. D. Becke, *J. Chem. Phys.*, 1993, **98**, 5648-5652.
3. C. Lee, W. Yang and R. G. Parr, *Phys. Rev. B*, 1988, **37**, 785-789.
4. W. J. Hehre, R. Ditchfield and J. A. Pople, *J. Chem. Phys.*, 1972, **56**, 2257-2261.
5. D.-L. Ma, T.-S. Lai, F.-Y. Chan, W.-H. Chung, R. Abagyan, Y.-C. Leung and K.-Y. Wong, *ChemMedChem*, 2008, **3**, 881-884.
6. M. Totrov and R. Abagyan, *Proteins: Structure, Function, and Bioinformatics*, 1997, **29**, 215-220.
7. T.-M. Ou, Y.-J. Lu, C. Zhang, Z.-S. Huang, X.-D. Wang, J.-H. Tan, Y. Chen, D.-L. Ma, K.-Y. Wong, J. C.-O. Tang, A. S.-C. Chan and L.-Q. Gu, *J. Med. Chem.*, 2007, **50**, 1465-1474.
8. P. Wu, D.-L. Ma, C.-H. Leung, S.-C. Yan, N. Zhu, R. Abagyan and C.-M. Che, *Chem. Eur. J*, 2009, **15**, 13008-13021.
9. D. Hayes, G. B. Griffin and G. S. Engel, *Science*, 2013, **340**, 1431-1434.
10. Y.-Y. Zhang, J.-L. Mi, C.-H. Zhou and X.-D. Zhou, *Eur. J. Med. Chem.*, 2011, **46**, 4391-4402.
11. D. Preti, P. G. Baraldi, G. Saponaro, R. Romagnoli, M. Aghazadeh Tabrizi, S. Baraldi, S. Cosconati, A. Bruno, E. Novellino, F. Vincenzi, A. Ravani, P. A. Borea and K. Varani, *J. Med. Chem.*, 2015, **58**, 3253-3267.
12. M. S. Lowry, W. R. Hudson, R. A. Pascal and S. Bernhard, *J. Am. Chem. Soc.*, 2004, **126**, 14129-14135.
13. A. Renaud de la Faverie, A. Guédin, A. Bedrat, L. A. Yatsunyk and J.-L. Mergny, *Nucleic Acids Res.*, 2014, **42**, e65.
